# AAPM‐HPS medical physics practice guideline 16.a: medical health physics scope of practice

**DOI:** 10.1002/acm2.70734

**Published:** 2026-10-06

**Authors:** Kenneth Lewis, John Metyko, Jessica Clements, Marc Felice, Mary Ellen Jafari, Melissa Martin, Thomas Morgan, Glenn Sturchio, Chu Wang, Neil Whiteside

**Affiliations:** ^1^ Radiology and Radiological Sciences Vanderbilt University Medical Center Nashville Tennessee USA; ^2^ American Association of Physicists in Medicine Representative Alexandria Virginia USA; ^3^ University of Texas MD Anderson Cancer Center Houston Texas USA; ^4^ Health Physics Society Representative Herndon Virginia USA; ^5^ University of Vermont Medical Center Burlington Vermont USA; ^6^ University of Pennsylvania Philadelphia Pennsylvania USA; ^7^ Kaiser Permanente Southern California Medical Group Pasadena California USA; ^8^ Therapy Physics, Inc. Signal Hill California USA; ^9^ Versant Medical Physics and Radiation Safety Sarasota Florida USA; ^10^ Mayo Clinic Jacksonville Florida USA; ^11^ Duke University Durham North Carolina USA; ^12^ Yale University New Haven Connecticut USA

**Keywords:** medical health physics, MPPG, practice guideline, scope of practice

## Abstract

The field of Medical Health Physics (MHP) lies at the intersection of Health Physics (HP) and Medical Physics (MP). The MHP is one of the five subfields identified by the AAPM that in which a Qualified Medical Physicist (QMP) may operate independently to provide clinical professional services. The pathway to become a QMP employed in the field of MHP varies depending on which community an individual originates from (HP or MP). This document aims to identify the minimum educational requirement, appropriate professional certification(s), work experience, and the minimum areas of responsibility (scope of practice) for a QMP working the MHP subfield.

## ABOUT MEDICAL PHYSICS PRACTICE GUIDELINES

The American Association of Physicists in Medicine (AAPM) is a nonprofit professional society whose primary purposes are to advance the science, education, and professional practice of medical physics. The AAPM has roughly 10,000 members and is the principal organization of medical physicists in the United States.

The AAPM will periodically define new practice guidelines for MP practice to help advance the science of MP and to improve the quality of service to patients throughout the United States. Existing MP practice guidelines will be reviewed for the purpose of revision or renewal, as appropriate, on their fifth anniversary or sooner.

Each MP practice guideline represents a policy statement by the AAPM, has undergone a thorough consensus process in which it has been subjected to extensive review, and requires the approval of the Professional Council. The MP practice guidelines recognize that the safe and effective use of diagnostic and therapeutic radiology requires specific training, skills, and techniques, as described in each document. Reproduction or modification of the published practice guidelines and technical standards by those entities not providing these services is not authorized.

The following terms are used in the AAPM practice guidelines:

- Must and Must Not: Used to indicate that adherence to the recommendation is considered necessary to conform to this practice guideline. **While must is the term to be used in the guidelines, if an entity that adopts the guideline has shall as the preferred term, the AAPM considers that must and shall have the same meaning**.
- Should and Should Not: Used to indicate a prudent practice to which exceptions may occasionally be made in appropriate circumstances.

## 1 INTRODUCTION

The field of Medical Health Physics (MHP) lies at the intersection of Health Physics (HP) and MP. In general, it can be described as that area of HP that is concerned with the protection of healthcare workers, members of the public, and patients from unwarranted radiation exposure received in a medical environment.^1^

A Qualified Medical Physicist (QMP) must be knowledgeable in the principles of radiation protection and familiar with the variety of radiation applications in medicine.^1^, ^2^ This document describes the qualifications necessary for a QMP with responsibilities as a radiation safety professional in the MHP field, along with the associated general duties/responsibilities, which are detailed in the appendix table. A QMP is defined by AAPM Position Policy and Statements and has specific roles described in numerous ACR‐AAPM Practice Parameters and Technical Standards documents.^2^, ^3^, ^4^

This document will not address the QMP's role in performing prospective internal dosimetry calculations for patients undergoing radiopharmaceutical therapy (RPT).

### 1.1 Background

In the early days of medical radiation use, radiation protection practices were limited, inadequate, and unevenly applied by the medical community. Professional and public literature are filled with unfortunate incidents where radiation caused harm instead of benefit.^5^, ^6^, ^7^, ^8^, ^9^ Following the development of the MP and HP fields, medical radiation protection evolved into a formal discipline. MHP is now considered a sub‐specialty of both the MP and HP professions.

## 2 INTENDED USERS

The contents of this document can aid a QMP when determining an overall scope of practice for the performance of MHP activities. It may also be referenced by administrators, regulators, and accreditation bodies to identify the necessary qualifications for individuals performing MHP activities. This document, like all AAPM MP Practice Guidelines, will be revised at intervals not to exceed 5 years. Where these guidelines are adopted by other organizations, they must be incorporated by reference to reflect any updates made to this practice guideline. This document discusses the typical duties and minimum level of responsibility/involvement a QMP should provide.

## 3 QUALIFICATIONS

Currently, the training and experience pathway to become a QMP working in the field of MHP varies depending on which community an individual originates from—the MP community is typically more concerned with the application of physics in medicine, whereas the HP community is generally more concerned with radiation protection. All pathways require that QMPs possess a graduate level degree, specialty board certification, and at least 3–6 years of relevant professional experience.^2^, ^3^

### 3.1 Education

Due to the complexity and rapid growth of radiation use in medicine and the healthcare industry, it is highly recommended that all future medical health physicists possess a graduate degree in either HP, MP, or a closely related field.

### 3.2 Board Certification

Healthcare professionals are often required to be certified by a specialty board of examiners. Board certification establishes minimum standards for educational, ethical, and clinical training and a minimum competency level for professional practice. This aids in comparing and assessing professional competency due to different training and experience pathways.

For the health physicist, the American Board of Health Physics (ABHP) offers the Certified Health Physicist (CHP) certification. This credential indicates that the diplomate is proficient in applying radiation protection principles and techniques across a wide variety of radiation industries (e.g., nuclear power, environmental, research, medical, etc.).^10^ The CHP is a radiation protection professional generalist, though they typically possess specialized experience in one or more radiation industry fields.

In contrast, clinical medical physicists operate exclusively in the medical domain and are highly specialized in a particular subfield of MP.^2^ Each subfield has a separate board certification process. The American Board of Radiology (ABR) oversees certification for medical physicists in the fields of Therapeutic MP, Diagnostic MP, and Nuclear MP, and is the primary certification pathway for most medical physicists. Two additional organizations also provide sub‐specialty certification; these are: the American Board of Medical Physics (ABMP), and the American Board of Science in Nuclear Medicine (ABSNM). With respect to the MHP field, the ABMP offers a certification in MHP, while the ABSNM offers certifications in (1) Radiation Protection and (2) Nuclear Medicine Physics and Instrumentation.

### 3.3 Experience

Unlike many health care professions, the MHP field does not possess a dedicated residency training program. Due to the broad range of medical radiation applications, it can often take many years beyond the minimum board certification requirements to gain applicable experience in all medical radiation use areas. Thus, it is incumbent upon the potential QMP to only practice within their areas of expertise, or under the supervision of an expert in said field.

### 3.4 Non‐ionizing radiation

All QMPs have professional training in the safe use of ionizing radiation, but experience with non‐ionizing radiation is highly variable. Nonetheless, laser safety and MRI safety oversight are often expected of the QMP. This is especially common at larger healthcare institutions where the QMP is the institutional Laser Safety Officer (LSO)^11^ or the institutional Magnetic Resonance Safety Officer (MRSO),^12^, ^13^ while nurses or technologists serve as Deputy LSOs or Deputy MRSOs at the department level. Although a medical physicist traditionally fills the role of a Magnetic Resonance Safety Expert (MRSE),^12^, ^13^ at some institutions, a QMP (health physicist or medical physicist) serving as the medical health physicist may fill the institutional MRSO role, which is more of an administrative safety officer role with oversight over MR safety.

It is important that the QMP obtain the necessary training and board certification to fill any voids in their own training and experience pathway. This Medical Physics Practice Guideline (MPPG) does not address the specific requirements for non‐ionizing radiation safety training and experience. The non‐ionizing radiation safety tasks listed in the Appendix MHP Task List are only applicable for non‐ionizing radiation programs in which a QMP is involved. Smaller healthcare settings may rely entirely on physicians, nurses, and technologists for non‐ionizing radiation safety program oversight.

## 4 CONCLUSION

QMPs serve a highly specialized role in healthcare: they provide ionizing radiation protection support to a wide variety of medical services and are often enlisted to support non‐ionizing radiation safety programs in laser and magnetic resonance safety. Traditionally, they occupy the position of medical radiation safety officer and/or laser safety officer. Medical physicists and health physicists are both extensively trained to fulfill these roles, though their training pathways differ. Professional certification is essential to the QMP as it provides evidence of minimal competency to operate independently in the MHP field. The scope of responsibilities detailed in this document is by no means exhaustive—as a designated medical radiation safety officer will encompass most of these duties as part of regulatory responsibilities. However, for those QMPs that are not specifically identified on a radioactive materials license, this document can assist in identifying areas where a QMP can be of service. Note that there may be some responsibility overlap with MPPG Report 10.b—AAPM MP practice guideline 10.b.: Scope of practice for clinical MP.^4^

## 5 DEFINITIONS AND ABBREVIATIONS

### 5.1 Definitions

Health Physics (HP)–the branch of physics or biophysics concerned with radiation physics and radiation biology, which deals with the safe use of ionizing and nonionizing radiation in academic, government, industrial, medical, nuclear power, research, and other civilian and military applications of radiation.

Laser Safety Officer (LSO)–a designated individual responsible for the safe use of lasers in a work environment and who establishes proper control measures appropriate for different levels of laser hazards. (ANSI standard ANSI Z136.1 “Safe Use of Lasers”)

Medical Health Physics (MHP)–the area of health physics that is concerned with the protection of healthcare workers, members of the public, and patients from unwarranted radiation exposure received in a medical and research environment.^14^

Medical Physics (MP)–that branch of physics that is associated with the practice of medicine. The term MP, as it is used here, includes diagnostic MP, therapeutic medical physics, nuclear medical physics, and MHP.

Magnetic Resonance Safety Expert (MRSE)–An individual with significant technical knowledge of the underlying physics and safety aspects of MRI who also serves as a resource/advisor to the Magnetic Imaging Medical Director (MRMD) and/or Magnetic Resonances Safety Officer (MRSO). This role is normally held by a medical physicist with the American Board of Magnetic Resonance Safety (ABMRS12) MSRE certification.

Magnetic Resonance Safety Officer (MRSO)–An individual designated responsible for the safety of staff and patients within an MRI suite. This responsibility typically is held by an experienced MRI technologist with an American Board of Magnetic Resonance Safety (ABMRS12) MSRO certification; however, some organizations may task the QMP to be the institutional administrative MRSO—with responsibilities varying depending on the institution.

Qualified Medical Physicist (QMP)–In the field of medical physics, the AAPM defines a Qualified Medical Physicist (QMP) as a clinical professional who is competent to independently provide clinical professional services in one or more of the five subfields of medical physics.^2^, ^3^

Radiation–includes both ionizing and nonionizing radiation in the forms of electromagnetic radiation and particulate radiation. The source of radiation could be associated with a radiological procedure, general medical device, or any non‐medical, occupational source of radiation.

### 5.2 Acronyms

AAHP: American Academy of Health Physics (AAHP)
ABHP: American Board of Health Physics (ABHP)
ABMP: American Board of Medical Physics (ABMP)
ABR: American Board of Radiology (ABR)
ABSNM: American Board of Science in Nuclear Medicine (ABSNM)
ACR: American College of Radiology
ALARA: As Low As Reasonably Achievable
AMP: Authorized Medical Physicist
AU: Authorized User
CHP: Certified Health Physicist
DCGL: Derived Concentration Guideline Level
DOT: Department of Transportation
ED: Emergency Department
HPS: Health Physics Society (HPS)
LLD: Lower Limit of Detection
LSO: Laser Safety Officer
MARSSIM: Multi‐Agency Radiation Survey and Site Investigation Manual
MDA: Minimal Detectable Activity
MPPG: Medical Physics Practice Guidelines
MRSE: Magnetic Resonance Safety Expert
MRSO: Magnetic Resonance Safety Officer
QA: Quality Assurance
QC: Quality Control
QMP: Qualified Medical Physicist
RAM: Radioactive Material
RSC: Radiation Safety Committee

## AUTHOR CONTRIBUTIONS

This guideline was developed by the Medical Physics Practice Guideline Task Group 386 of the Professional Council of the American Association of Physicists in Medicine. TG386 included members from both AAPM and the Health Physics Society. Each author reviewed recent literature on the topic and offered opinions on and language for the guideline. They also reviewed and applied comments from the full AAPM membership and HPS representatives to the document.

## CONFLICT OF INTEREST STATEMENT

The Chairs of Medical Physics Practice Guideline 16.a: Medical Health Physics Scope of Practice have reviewed the required Conflict of Interest statement on file for each member of TG386 and determined that disclosure of potential Conflicts of Interest is an adequate management plan.

The members of TG386 listed below attest that they have no potential Conflicts of Interest related to the subject matter or materials presented in this document: Kenneth Lewis, John Metyko, Jessica Clements, Marc Felice, Mary Ellen Jafari, Melissa Martin, Thomas Morgan, Glenn Sturchio, Chu Wang, Neil Whiteside.

## SCOPE OF PRACTICE COMBINED TASK LIST

The table below categorizes the areas/tasks that commonly fall under the responsibility of the QMP fulfilling the role of a medical health physicist and the recommended degree to which the physicist should be involved. It is understood that this task list is not exhaustive as it is likely that many QMPs have additional medical health physics responsibilities not included here. If a QMP does not have experience in a particular area or task for which they are responsible, it is expected that the QMP will obtain further training to become competent.

For tasks categorized as an activity that must be performed or supervised by QMP, the recommended level of supervision is specified in parentheses. The supervision terms are listed below as defined in AAPM medical physics practice guideline 3.b.: Levels of supervision for medical physicists in clinical training.^15^

“General Supervision—The procedure [or activity] is performed under a QMP's overall direction and control, but the QMP's presence is not required during the performance of the procedure [or activity]. Under General Supervision, the training of the personnel who actually perform the procedure and the maintenance of the necessary equipment and supplies are the continuing responsibility of the QMP.^15^”

“Direct Supervision—A QMP must exercise General Supervision and be present in the facility and immediately available to furnish assistance and direction throughout the performance of the procedure [or activity]. It does not mean that the QMP must be present in the room when the procedure [or activity] is being performed.^15^”

“Personal Supervision—A QMP must exercise General Supervision and be present in the room during the performance of the procedure [or activity].^15^”

**Table:**

Task Description (General)	Sub‐Task(s)	Activity MUST be performed by QMP	Activity must be performed or supervised by QMP	Activity SHOULD include a QMP	Amplifying Information
**ALARA planning**	Assess risk of exposure for work activities	x			
	Identify those with the potential for highest exposure		x (general)		
	Design work plans to achieve and maintain doses ALARA commensurate with work objectives and benefits to be gained		x (general)		
	Consider decreased worker efficiency from use of respiratory protective devices and/or heat stress mitigation equipment		x (general)		
	Evaluate the feasibility of using remote technologies to reduce occupational exposure			x	
**Audits/Inspections**	Perform testing of medical imaging equipment & safety (including dental)		x (general)		Report out to appropriate QMP subspecialist if necessary.
	Audits reports of medical imaging equipment & safety inspections/surveys			x	Report out to appropriate QMP subspecialist if necessary.
	Audit records of therapy equipment/programs			x	Report out to appropriate QMP subspecialist if necessary.
	Ensure AUs’ credentials are current		x (general)		
	RAM receipt surveys		x (general)		
	Verification of activity administered to patients		x (general)		
	Written directives		x (general)		
	10 CFR 37 source security records and processes		x (direct)		
**Certification of decommissioning of spaces and equipment removed from clinical use**		x			
**Committee membership**	Radiation Safety Committee	x			
	Laser Safety Committee		x (personal but may attend meetings virtually)		
	Magnetic Resonance Safety Committee			x	
	Radioactive Drug Research Committee	x			
	Institutional Review Board or subcommittee of RSC that reviews human research protocols	x			
**Collaboration with project managers for work performed on/in radiation areas**		x			
**Decommissioning: Implementation of Multiagency Radiation Survey and Site Investigation Manual (MARSSIM) processes and procedures**	Design, conduct, and report Site Characterization Survey	x			
	Identify isotopes contributing to contamination		x (general)		
	Calculate derived concentration guideline levels (DCGLs)	x			
	Develop plan to remove contamination below DCGLs		x (general)		
	Supervise cleanup work and survey site to ensure remaining contamination, if any, is below DCGLs		x (direct)		
**Compliance with laws, regulations, and policies and procedures**	Support regulatory inspections			x	Depends on the nature of the inspection or accreditation.
	Support hospital accreditation programs			x	
**Financial assurance for decommissioning**	Review 10 CFR 30.35 or Agreement State regulations to determine if financial assurance is required	x			
	Identify isotopes and locations/sites where requirements must be implemented	x			
	Assist with estimating decommissioning costs in accordance with requirements of MARSSIM	x			
**Instrumentation**					
**(non‐imaging equipment, e.g., meters, counters, surgical gamma probes, etc.)**	Instrument selection		x (general)		
	Measurement interpretation		x (general)		
	Design of action plans based on instrument readings		x (general)		
	Calibrations		x (general)		
	Repairs		x (general)		
	Efficiencies		x (general)		
	LLDs or MDAs		x (general)		
	QA/QC		x (general)		
**Medical events and corrective actions**	Conduct investigation, including Joint Commission sentinel events		x (general)		
	Recommend corrective and preventative actions		x (general)		
**Triage and management of mass casualties admitted to an Emergency Department (ED)**	Train ED staff in radiation effects and handling contaminated patients		x (general)		
	Advise ED staff on testing and treatment options		x (general)		
**Management of radiation producing devices**	Manage registration of ionizing radiation equipment		x (general)		
	Oversee performance testing program		x (general)		
**Radiation Protection Program Management**		x			
**Radioactive Materials: Radioactive waste management**	**Radiation surveys**				
	Measure ambient radiation dose rates		x (general)		
	Perform area survey		x (general)		
	Assess removable contamination		x (general)		
	**Radioactive cadaver management**				
	Identify isotope		x (general)		
	Interpret dose rate readings		x (general)		
	Notify funeral homes and regulatory agencies as required	x			
**Radioactive Materials: Licensing**	Draft applications and amendments		x (general)		
	Interpret license limitations and requirements for users and administrators	x			
	Ensure operating policies and procedures are in compliance with license requirements		x (general)		
**Radioactive Materials: Mammography /**					
**Breast Imaging**	Oversee radioactive seed program			x	Task performed by qualified individuals, report out to MHP QMP if necessary.
	Train Authorized Users		x (general)		
	Train and observe surgeons' use of radioactive seeds		x (direct)		
	Manage and track misplaced seeds		x (direct)		
	Develop and implement emergency response procedures	x			
**Radioactive Materials: Safety and Security**	Manage sealed source inventory and leak testing		x (general)		
	Manage training of users		x (general)		
	Manage 10 CFR 37 requirements		x (general)		
	Develop and implement policies and procedures		x (general)		
	Ensure background checks are performed		x (general)		
	Develop and oversee physical security requirements		x (general)		
	Identify individuals who require training and approve training materials and methods		x (general)		
	Develop processes for and oversee transportation and source replacement		x (direct)		
	Coordinate table‐top exercises		x (direct)		
	Liaise with local law enforcement agencies		x (general)		
	Liaise with local first responders		x (general)		
	Manage media relations			x	
	Oversee reciprocity inspections		x (direct)		
**Assessment of doses to members of the public**			x (general)		
**Dosimetry External**	Manage radiation badges		x (general)		
	Perform ALARA investigations		x (general)		
	Develop and implement radiation protection solutions		x (general)		
	Oversee inventory and inspection of protective garments (aprons, shields, thyroid collars)		x (general)		
	Oversee pregnancy declarations		x (general)		
	Oversee prior occupational dose history reports		x (general)		
	Perform and oversee dose summations (multiple employers in calendar year)		x (general)		
	Perform risk assessments		x (general)		
**Dosimetry Internal**	Oversee bioassay programs	x			
	Oversee fetal dose evaluations	x			
	Determine post‐administration compliance during intake assessment	x			
	Perform risk assessments	x			
**Non‐Ionizing Radiation: RF & MRI Safety**				x	See MMPG 10 for additional non‐ionizing radiation MRI safety items
**Non‐Ionizing Radiation: Laser Safety**	Perform annual audit		x (general)		
	Manage authorizations of laser use		x (general)		
	Assess patient/staff injury			x	
	Develop and oversee laser safety education		x (general)		
	Inspect protective eyewear		x (general)		
	Perform audits		x (general)		
	Inspect devices		x (general)		
	Competency		x (general)		
	Manage medical practitioner privileging for laser use			x	
	Perform inventory inspections		x (general)		
	Ensure appropriate safety signage is posted		x (general)		
	Manage laser safety program		x (general)		
	Assess loaner, rental, and demo units		x (general)		
**Radiation Protection: Patient release post‐treatment with radiopharmaceuticals**	Measure post‐treatment activity		x (general)		
	Calculate dose rates		x (general)		
	Develop discharge instructions and ensure appropriate dissemination		x (general)		
**Radiation Protection**:					
**Use of unsealed sources of radioactive material**	Oversee use in clinical nuclear medicine departments, research laboratories, and animal research facilities		x (general)		
	Develop and implement emergency response procedures		x (general)		
	Develop processes for managing and responding to radioactive spills		x (general)		
	Ensure rooms used for patients treated with radiopharmaceuticals are properly prepared and decontaminated		x (general)		
**Radiation Protection: Shielding calculations and evaluations**	Determine the type and amount of shielding required	x			
	Assess construction plans	x			
	Assess shielding integrity	x			
**Radiation Safety**:					
**Medical research studies**	Calculate radiation doses to subjects		x (general)		
	Calculate risk estimates		x (general)		
**Education**:					
**Radiation Safety Training**	Provide initial and annual training		x (general)		
	Provide training for the safe use of ionizing radiation devices		x (general)		
	Provide information about Image Gently, Wisely, and ALARA		x (general)		
	Perform US Department of Transportation (DOT) required training for transporting radioactive materials		x (general)		
	Coordinate with and provide education for first responders		x (general)		
	Provide education for residents		x (general)		
**Misc**.	Implement new services involving ionizing radiation or lasers	x			
	Evaluate emerging radioactive treatments	x			
	Manage animals administered with radioactive materials		x (general)		
	Manage security of radioactive material		x (general)		
	Manage determinations and permitting of effluent air and water		x (general)		
	Ensure and verify safety postings		x (general)		
	Oversee radiation protection in Pathology and Laboratory Medicine		x (general)		
	Manage intake of radioactive sources		x (general)		
	Oversee personnel and patient exposures to radiation		x (general)		
	Oversee exposures to pregnant staff and patients		x (general)		
	Address radiation safety concerns of staff, patients, and families		x (general)		
